# Calcium Alginate Gels as Stem Cell Matrix – Making Paracrine Stem Cell Activity Available for Enhanced Healing after Surgery

**DOI:** 10.1371/journal.pone.0118937

**Published:** 2015-03-20

**Authors:** Andreas Schmitt, Philipp Rödel, Cihad Anamur, Claudine Seeliger, Andreas B. Imhoff, Elmar Herbst, Stephan Vogt, Martijn van Griensven, Gerhard Winter, Julia Engert

**Affiliations:** 1 Department of Pharmacy, Pharmaceutical Technology and Biopharmaceutics, Ludwig-Maximilians-University Munich, Butenandtstr. 5, Haus B, D-81377 Munich, Germany; 2 Department of Sports Orthopedics, Technical University Munich, Ismaninger Str. 22, D-81675 Munich, Germany; 3 Department of Trauma Surgery, Technical University Munich, Ismaninger Str. 22, D-81675 Munich, Germany; 4 Department of Trauma Surgery, Medical University Innsbruck (MUI), Anichstr. 35, A-6020 Innsbruck, Austria; University of Sheffield, UNITED KINGDOM

## Abstract

Regeneration after surgery can be improved by the administration of anabolic growth factors. However, to locally maintain these factors at the site of regeneration is problematic. The aim of this study was to develop a matrix system containing human mesenchymal stem cells (MSCs) which can be applied to the surgical site and allows the secretion of endogenous healing factors from the cells. Calcium alginate gels were prepared by a combination of internal and external gelation. The gelling behaviour, mechanical stability, surface adhesive properties and injectability of the gels were investigated. The permeability of the gels for growth factors was analysed using bovine serum albumin and lysozyme as model proteins. Human MSCs were isolated, cultivated and seeded into the alginate gels. Cell viability was determined by AlamarBlue assay and fluorescence microscopy. The release of human VEGF and bFGF from the cells was determined using an enzyme-linked immunoassay. Gels with sufficient mechanical properties were prepared which remained injectable through a syringe and solidified in a sufficient time frame after application. Surface adhesion was improved by the addition of polyethylene glycol 300,000 and hyaluronic acid. Humans MSCs remained viable for the duration of 6 weeks within the gels. Human VEGF and bFGF was found in quantifiable concentrations in cell culture supernatants of gels loaded with MSCs and incubated for a period of 6 weeks. This work shows that calcium alginate gels can function as immobilization matrices for human MSCs.

## Introduction

Recent research has focused on improvement of the healing capacity of various tissues after surgery. Here the application of anabolic (e.g. bFGF, IGF, TGFβ1) and proangiogenic growth factors (e.g. VEGF) resulted in improvement of regenerate quality and strength in different animal models [1,2,3,4,5]. However, due to the low stability of the growth factors either multiple injections of recombinant proteins or stable gene transfer was necessary to achieve these results. Due to safety reasons gene transfer is presently not applicable in patients. Furthermore, the necessity of repetitive local injections would cause enormous costs and considerable burden for the patient with an increased infection risk. Hence, none of these treatments has yet reached patient therapy.

During the last decade, autologous mesenchymal stem cells (MSCs) have received more and more interest within the field of regenerative medicine. These adult stem cells are easy to harvest and have the potential to differentiate into mesenchymal cell types, such as tenocytes, chondrocytes and osteoblasts, hence making them a promising tool in mesenchymal tissue regeneration. Several studies have revealed beneficial effects of MSCs on tissue regeneration in animals [6]. Here, MSCs participated in the healing process and differentiated into local tissue cells leading to better regenerates [7]. Furthermore, recent studies revealed that the most important impact of MSCs on tissue regeneration is most likely their paracrine activity. Upon secretion of a cocktail of anabolic cytokines, healing mechanisms are improved. This important paracrine activity recently even caused some authors to call MSCs an “injury drug store” [8].

The aim of our present study was to establish a delivery system that makes the paracrine activity of autologous mesenchymal stem cells usable to enhance regeneration after surgery. The goal of the project was to establish a method which is applicable during arthroscopic and open surgical procedure and directly transferable to the operation theatre. Therefore, we designed a matrix as a carrier that allows immobilization of autologous MSCs harvested during operation. The matrix has to fulfil several properties: it should promote survival of the incorporated cells for at least 6 weeks (which is the average time span for regeneration of most tissues), while at the same time it should allow for the diffusion of growth factors from the matrix into the environment. Additionally, the matrix should be readily applicable during open and arthroscopic surgeries. Finally, the matrix should show adhesion to collagen to allow anchoring of the matrix on the host tissue, should be injectable using a standard syringe and should solidify within 30 minutes during surgery.

Within the present study, alginate hydrogels were chosen as basis of matrix, due to its suitable mechanical properties and proven biocompatibility. Alginate hydrogels were systematically modified towards the desired requirements by optimisation of the gelation process, alginate concentration and addition of hyaluronic acid and polyethylene glycol 300,000. Suitability of the obtained hydrogels was proven *in vitro* using primary human MSCs.

## Materials and Methods

### Materials

Sodium alginate Biochemica was obtained from AppliChem GmbH, Darmstadt, Germany. Alginic acid sodium salt from brown algae was obtained from Sigma-Aldrich, Taufkirchen, Germany. DMEM high glucose (4.5 g/L), fetal calf serum, Pen/Strep-PreMix and Trypsin/EDTA, and LSM 1077 Medium were purchased from PAA Laboratories, Pasching, Austria. CalceinAM, Hoechst 33342, bovine serum albumin, ABTS and Dulbecco’s phosphate buffered saline without calcium/magnesium were obtained from Sigma-Aldrich, Taufkirchen, Germany. AlamarBlue was purchased from Biozol, Eching, Germany. Lysozyme was purchased from Dalian Greensnow Egg Products Development Co., LTD, Dalian, China. Hyaluronic acid for pharmaceutical production with an intrinsic viscosity of 2.7 m^3^/kg obtained from fermentation from *Streptococcus Zooepidemicus* was purchased from Shiseido Co. Ltd, Tokyo, Japan. Polyethylene glycol (PEG; MW 300,000 Da) was obtained from Sigma-Aldrich, Taufkirchen, Germany. All other chemical were of analytical grade.

### Methods

#### Preparation of alginate gels

Sodium alginate Biochemica (alginate 1) and Alginic acid sodium salt from brown algae (alginate 2) (Sigma-Aldrich, Germany) were prepared as 2.0% (w/w) solutions and dispersed in 150 mM phosphate buffered saline (PBS) pH 7.0 and stirred in a glass beaker overnight at room temperature. In addition, PEG or sodium hyaluronate (NaHa) and combinations thereof were used to prepare alginate hybrid gels. After the alginate was completely dissolved, PEG or NaHa were added at their respective concentrations (w/w) and stirred again overnight until a homogenous viscous alginate solution was obtained. Non-sterile conditions were used for the preparation of the gels for studying the gel composition and mechanical stability. For the cell assays, all solutions were sterile-filtered using 0.2 μm filters (Aerodisc LC 25 mm, PALL Life Sciences, Ann Arbor, MI, USA) prior to the gel preparation.

#### Internal gelation

5 ml calcium carbonate and D-glucono-delta-lacton (GDL) suspensions in water were prepared at 1:2 ratios and concentrations ranging from 5 mM to 200 mM and 10 mM to 400 mM, respectively, using cold, highly purified water (5°C). 15 ml of the alginate solution described above were added into a 60 ml flat bottom falcon tube. Subsequently, 5 ml of the CaCO_3_ and GDL suspension were added and vortexed for 30 seconds.

#### External gelation

15 ml of the alginate solution described above were added into a 60 ml flat bottom falcon tube. Subsequently, 5.0 ml of a calcium chloride solutions ranging from 0 to 300 mM were carefully added to the tube on the rim of the falcon tube to cover the alginate and induce external gelation.

#### Combination of internal and external gelation

Sodium alginate solutions (alginate 1 or 2) (5.00 g± 0.05 g) and calcium carbonate/GDL suspensions (2.00 g ± 0.05 g) were weighed and added to a flat-bottom 60 ml falcon tube. The resulting mixture was vortexed (Merck KGaA, Darmstadt, Germany) for 30 seconds. The gels were then covered with 5.0 ml calcium chloride solution and left to equilibrate for 30 minutes. Subsequently, the gels were washed twice with 10 ml PBS and finally covered with 20 ml PBS and left for 2 hours at room temperature.

#### Determination of rheological properties and gelling behaviour

The rheological behaviour was investigated using a MCR 100 rheometer (Physica—Anton Paar, Graz, Austria) equipped with a cone plate setup and a CP50–1 cone. The viscosity was observed as a function of time and shear rate. Experiments were conducted at 5°C, 20°C, and 37°C. A shear rate γ of 1/s was employed at a measurement position of 0.1 mm. Measurements were performed for the duration of 10 seconds per measurement point for a total duration of 30 minutes. Experiments were indepentently repeated three times with different samples.

#### Determination of water uptake capacity of alginate hydrogels

Alginate gels were prepared using the combination of internal and external gelation as described above. After incubation in CaCl_2_, specimens were carefully placed on light-duty tissues wipers (VWR, Ismaning, Germany). Samples were weighed directly after preparation (t0) and transferred into a 50 ml falcon tube filled with 25 ml of DMEM high glucose. Samples were placed in an incubator (Certomat IS, Sartorius AG, Goettingen, Germany) at 37°C and 40 rpm. DMEM high glucose was exchanged daily. Weighing of the alginate gels was repeated on days 1, 7, 14, and 20 days after preparation to determine the uptake capacity of the alginate hydrogels.

#### Determination of mechanical stability (compressibility and stiffness)

**Fig 1:**
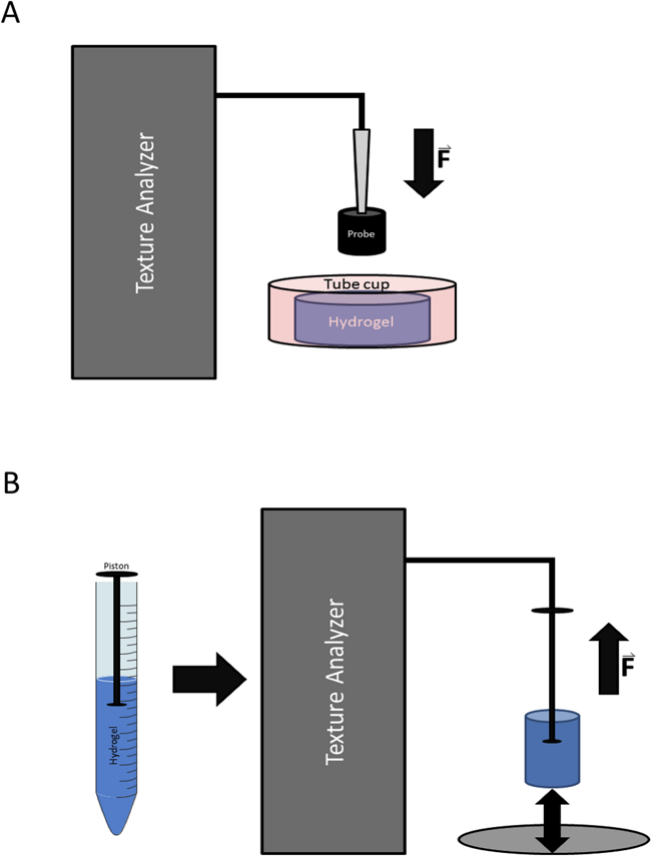
Schematic construction of the texture analyser. **(A) Compressibility and stiffness measurements using the texture analyser.** The falcon tube cap was used as a constant plane surface, for stabilisation and for central positioning of the sample during the measurement. **(B) Surface adhesion measurements using texture analyser.** A piston reversely embedded into the gel of a syringe served as connector to the texture analyser for adhesion measurements on three different surfaces (teflon, petri dish, gelatine gel).

A TA.XTplus Texture analyser (Stable Micro Systems, Surrey, UK) having the following technical specifications (Force Capacity: 50 kg.f (500 N); Force Resolution: 0.1 g; Loadcells: 0.5, 5, 30, 50 kg.f; Speed Range: 0.01–40 mm/s; Range Setting: 0.01–280 mm) was utilized to determine the mechanical stability of the gels. Gels were prepared in 60 ml falcon tube caps to provide a constant plane surface and to stabilize the central position of the samples during the measurement (Fig. 1a). A cylindrical probe with a circular area of 0.4 mm^2^ was used for all measurements. Measurements were performed at a test speed of 0.5 mm/s when a trigger force of 0.1 N was reached. Emerging forces were measured over a distance of 8.0 mm, which corresponds to half the gel height. For each formulation, three independent gels were measured. Data analysis was performed using the TextureExponent32 software (Stable Micro Systems, Surrey, UK). Gel strength was defined as the maximum force applied before rupture of the gels occurred.

For long-term mechanical stability, gels were prepared as described above with the addition of sodium azide to prevent bacterial growth. Samples were placed into a horizontal shaker (Heidolph Instruments, Schwabach, Germany) at 37°C at a shaking rate of 5 rpm. Samples were taken after 0, 1, 3 and 6 weeks, and the mechanical stability was tested on tree independent gels on each time point, as described above.

#### Determination of surface adhesion properties

Gels were prepared in a 15 ml Falcon tube into which a piston of a 1 ml syringe (Normject, Henke-Sass Wolf, Tuttlingen, Germany) had been inversely placed prior to the solidification process (Fig. 1b). After external gelation, gels were gently pulled from the falcon tube and cut with a sharp blade. The piston was then attached to a custom made holder for the texture analyser. Adhesion of the gels was measured on three different surfaces: Teflon, polystyrene and gelatin-gels. Alginate gels were pressed with a trigger force of 0.05 N and applied force of 0.5 N onto the respective surfaces for 10 seconds. The force required to detach the gels from the surface was measured as adhesion force. The test speed applied was 0.05 mm/s. Adhesion was tested on three independent gels for each formulation.

#### Injectability and spreading after injection

Calcium-alginate gels were filled into commercially available syringes (10/20 ml Normject, Henke-Sass Wolf, Tuttlingen, Germany) at 0, 5, 10, 15, 20, and 30 minutes after internal gelation had been initialized. The syringe was then attached to a custom made holder to the texture analyser, and the force to eject a volume of 5 ml from the syringe having an inner diameter of 2.3 mm was measured. Spreading of the gels after ejection was investigated macroscopically. Testing was repeated within three independent experiments.

#### Loading of gels with proteins

Bovine serum albumin (BSA) and lysozyme were used as model proteins to investigate loading and release from the gels. BSA (molecular weight 67 kDa, isoelectic point 4.5) served as a model for human vascular endothelial growth factor (VEGF), whereas lysozyme (molecular weight 11.7 kDa, isoelectic point 11.5) served as a model for human fibroblast growth factor 2 (h-bFGF). BSA and lysozyme were dissolved in PBS at a concentration of 7.0 mg/ml as determined spectroscopically using a NanoDrop2000 UV spectrophotometer (Thermo Scientific, Wilmington, USA) by using the extinction coefficient/molar absorptivity at 280 nm (2.65 mg^-1^ ml cm^-1^ for lysozyme; 1.346 mg^-1^ ml cm^-1^ for BSA). After the addition of the calciumcarbonate/GDL mixture, the final protein concentration was 5.0 mg/ml. Alginate gels were transferred into 60 ml falcon tubes containing 35 ml of 150 mM PBS buffer pH 7.4 and incubated in a horizontal shaker (Heidolph Instruments, Schwabach, Germany) at 37°C at 5 rpm for 168 hours. The falcon tubes were placed horizontally, so that the release media was able to completely cover the gels and there was no restriction as to the release from the gels. Testing was performed in three independent experiments.

#### Microscopic investigations

To determine the distribution and shape of calcium carbonate crystals in the gels, gels were cut into 1 mm thin slices using a microtome blade. The slices were subsequently investigated using an incident light microscope VHX-500 F (Keyence, Osaka, Japan).

#### Isolation of primary human bone marrow-derived mesenchymal stem cells (MSCs)

3–5 ml bone marrow was obtained from patients after their informed consent. The study was approved by the ethics committee of the faculty of medicine of the Technical University of Munich (Project Number 5217/111, TU Munich, Germany). Before bone marrow isolation, patients gave their written informed consent. Within the present study, we isolated MSCs from three patients (two female, age 80 and 55 years and one male 76 years). The following stem cell experiments were performed independently with cells from each patient.

Isolation was performed aseptically in a laminar air flow cabinet (Thermo Scientific, Langenselbold, Germany). Cell culture media was tempered in a water bath at 37°C prior to use. Bone marrow was diluted with sterile PBS until pipetting of the sample was possible. In a 15 ml Falcon tube a volume of 8 ml of the bone marrow samples were carefully transferred on 5 ml of lymphocyte separation medium LSM 1077. Tubes were centrifuged for 20 minutes at 650 x g without brake (Eppendorf, Hamburg, Germany) until samples were clearly separated into fat, serum, mononuclear cells, lymphocyte separation medium and erythrocytes and bone fragments. The layer containing the mononuclear cells was collected, washed with PBS and transferred to a 75 cm^2^ cell culture bottle to which 15 ml of cell culture media (DMEM high glucose 4.5 g/L, 10% bovine fetal calf serum and 1% penicillin/streptomycin (10000 units of penicillin, 1000 μg of streptomycin)) were added. Samples were incubated at 37°C and in a 5% CO_2_-atmosphere in an incubator (Thermo Electron LED, Langenselbold, Germany). After 48 hours, the medium was exchanged to remove non-adherent cells.

#### Cultivation of human bone marrow-derived MSCs

Cells were cultured in cell culture bottles containing 0.2 ml cell culture media per cm^2^. Media consisted of DMEM high glucose 4.5 g/L, 10% bovine fetal calf serum and penicillin/streptomycin (10000 units of penicillin, 1000 μg of streptomycin, 29.2 mg/ml L-glutamine in a 10 mM citrate buffer). Adherent cell culture was passaged after reaching approximately 70–80% confluence. Cells were detached from the bottle surface using 1.5 ml trypsin/EDTA. Addition of new media was used to stop the enzymatic reaction. Cells were cultivated for three passages before they were used for experiments. Before using the cells, cultures were routinely confirmed to be negative for CD 14, CD 45 and positive for CD90 and CD 105 by FACS analysis. Furthermore, multipotent differentiation potential towards osteogenic, chondrogenic and adipogenic lineage was confirmed for each used cell batch.

#### Seeding and cultivation of human bone marrow-derived MSCs into alginate gels

Aliquots of cell suspensions (3x10^6^ cells) were transferred into 50 ml Falcon tubes and centrifuged for 10 min at room temperature at 350 x g (Eppendorf, Hamburg, Germany). Subsequently, the supernatant was collected and discarded. A volume of 3 ml alginate solution was added to the tubes and vortexed for 30 seconds to suspend the cells within the viscous solutions. For the “internal gelation” a volume of 1.2 ml of calcium chloride/GDL suspension was added to the alginate-cell-mixture, followed by vortexing for 30 seconds. 3 ml of the suspension was pipetted into a six-well plate to which 4.0 ml of a 200 mM calcium chloride solution was added. All samples were left to equilibrate for 30 min at room temperature.

Alginate gels loaded with MSC cells were cultivated at 37°C in a 5% CO_2_ atmosphere. 3 ml of each gel was placed in a well of a six well plate filled with 9 ml of cell culture media. Media exchange was performed once per week. Supernatants were collected and stored at -80°C (Heraeus GmbH, Hanau, Germany) until they were analysed.

#### AlamarBlue assay for the determination of cell viability

To investigate cell viability within the alginate gels, we independently tested MSCs harvested from three donors. Cell culture media of cells entrapped in alginate gels was aspirated and 1 ml of a 10% AlamarBlue solution was added to each sample. Cells were then incubated at 37°C for 100 minutes. After incubation, three 100 μl samples of each Alamar blue solution were transferred to a NUNC maxisorp 96 well plate (Thermo Scientific, Wilmington, USA) and the absorption was measured at 600 nm and 570 nm using a FLUOstar OMEGA plate reader (BMG Labtech, Ortenberg, Germany). Alginate gels without cells served as negative control. Positive controls were obtained from subconfluent 2D cell cultures of MSCs in 6 well plates. Relative viability was calculated according to the manufacturer’s protocol. Blank for calculation was Alamar blue reagent without cells for 2D control, respectively Alamar blue reagent on Alginate gel without cells for gel testing.

#### Fluorescence microscopy

To the alginate gels loaded with MSC samples 6 μl of CalceinAM was added and plates were incubated for 30 minutes at 37°C. Next, 2 μl Hoechst 33342 at a concentration of 0.5 μg/l in PBS was added to each well and incubated at room temperature for 15 minutes. Cells were then analysed using a Keyence BZ-9000 fluorescence microscope (Keyence, Osaka, Japan). Photographs were combined by z-stacking. Viable and dead cells in the gels were observed. Gels loaded with cells from three donors were independently investigated.

#### Human VEGF and bFGF enzyme-linked immunoassay of cell culture supernatants

The following experiments were independently performed on MSCs harvested from three donors. Supernatants were collected during the six weeks of cell culture, immediately frozen at -80°C and stored until analysed. Human VEGF and bFGF Mini ELISA Development Kit, respectively, were purchased from Peprotech (Hamburg, Germany) and used as per manufacturer’s instructions. For the sandwich ELISA assay, 100 μl of capture antibody at a concentration of 0.50 μg/ml was incubated in each well over night at room temperature. Plates were washed four times and blocked and subsequently, 100 μl of standard (hVEGF or bFGF) or samples were added. Of each sample, three aliquots were tested. A biotin-labelled antibody was added at a concentration of 1 μg/ml. Plates were again washed four times and then 100 μl of a 1:2000 dilution of Avidin horse-radish peroxidase conjugate (avidin-HRP) was added and incubated for 30 minutes at room temperature. Finally, 100 μl of ABTS substrate was added and plates were measured at 405 nm and 650 nm using the FLUOstar Omega plate reader. Plates were measured at 5 minute intervals for the duration of 20 minutes.

#### Statistical analysis

For the statistical analysis, SPSS 20.0 (IBM SPSS Statistics, New York, USA) for Mac software was used. The normal distribution was tested and confirmed with the Kolmogorov-Smirnov test. The quantitative parameters were evaluated with the calculation of the mean and standard deviations (SDs). Depending on the data distribution the Student′s t test or Mann-Whitney U test and Wilcoxon test for unpaired and paired measurements were used. All the measurements are expressed with ± 1 SD. Statistical significance was accepted for *p* ≤ 0.05. p—values below 0.001 were considered as highly significant.

## Results

### Formulation of a hydrogel which meets the desired requirements

#### Solidification of the alginate gel within 30 minutes

To solve this problem, we decided to add a carrier for Ca^2+^ ions directly into the alginate matrix. A gelling mechanism with calcium carbonate (CaCO_3_) and D-glucono-δ-lacton (GDL) (internal gelation) has been described [9,10]. Here, the GDL is slowly hydrolysed, which results in a drop of the pH of the solution and subsequently a release of Ca^2+^ ions from the CaCO_3_ into the solution. We observed a time-delayed release of calcium ions, resulting in homogenous alginate gels. However, internal gelation alone also did not provide a gel that solidifies in the time frame of 30 minutes (data not shown).

**Fig 2:**
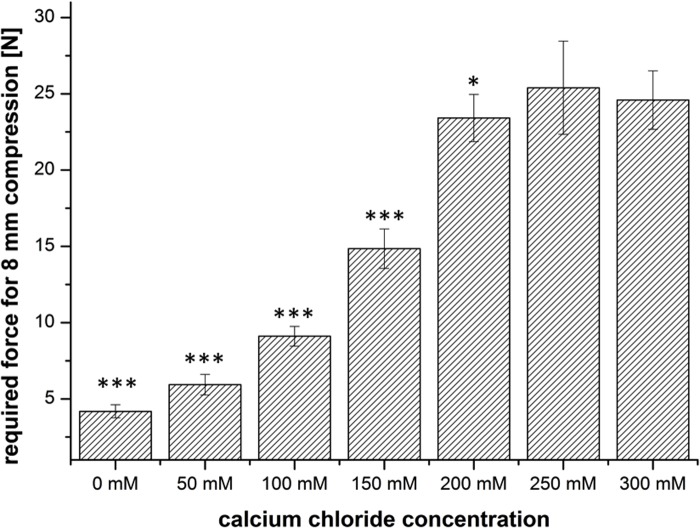
Mechanical stability of alginate gels upon external gelation using increasing concentrations of CaCl_2_. CaCl_2_-concentrations lower 200mM lead to significant lower mechanical stability compared to 300mM (n = 3 ± SD; * p< 0,05; **p<0,01; ***p<0,001).

**Fig 3:**
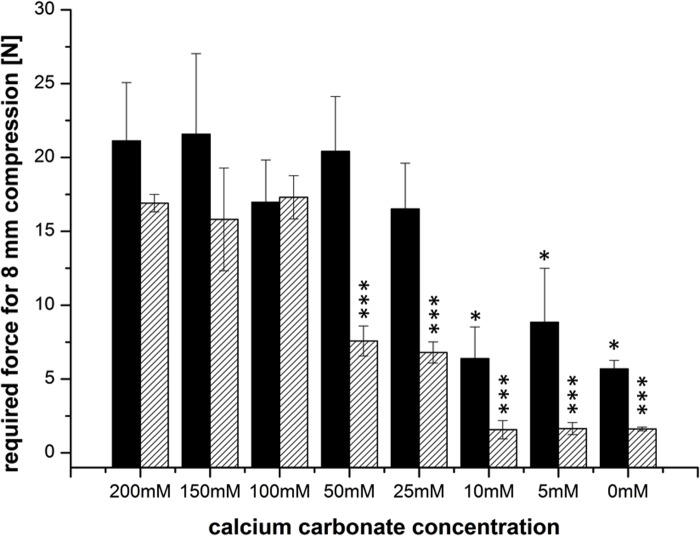
Mechanical stability of alginate gels upon internal gelation using varying concentrations of CaCO_3_. The black bar represents the required compression force directly after preparation, the dashed bar represents the required force after one week of aging. CaCO_3_-conzentrations lower 25mM lead to significant lower mechanical stability compared to full gelation with 200mM. After one week gels with 50mM CaCO_3_ and lower resulted in significant lower mechanical stability compared to full gelation with 200mM (n = 3 ± SD; * p< 0,05; **p<0,01; ***p<0,001).

**Fig 4:**
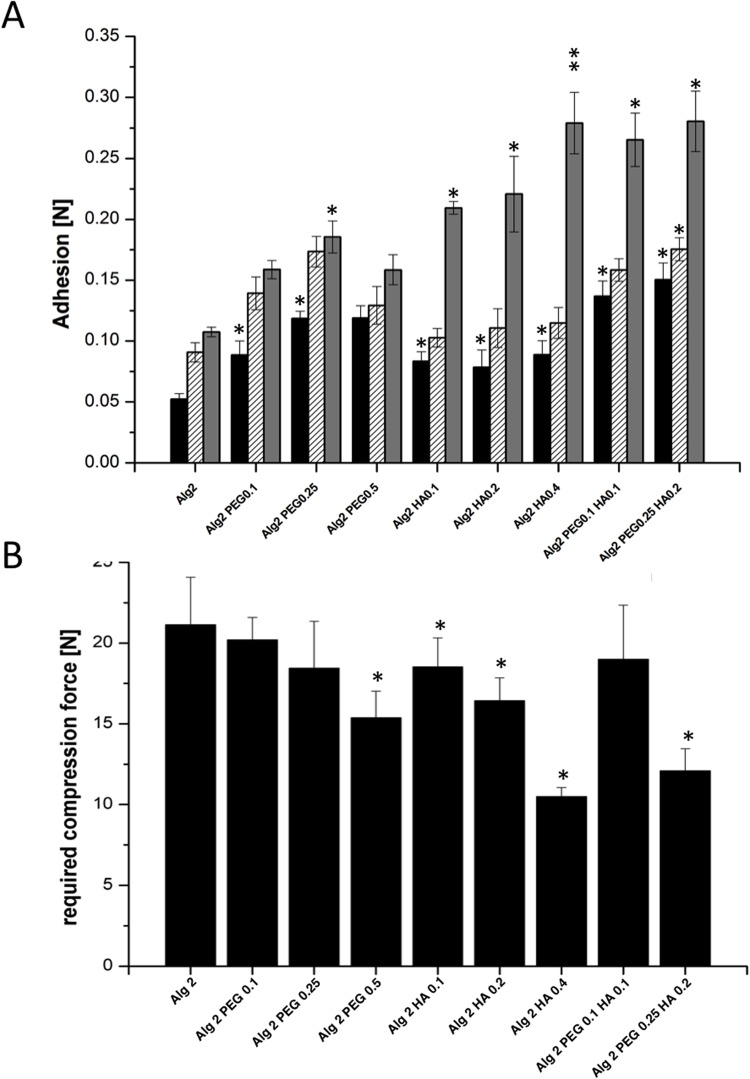
Mechanical testing of the alginate gels. **A) Adhesion of the different alginate and alginate hybrid gels on three different surfaces.** Addition of PEG and/or HA leads to significant increase in adhesion on certain surfaces compared to alginate gel without PEG or HA (Alg2). Black bar: Teflon, dashed bar: Polystyrene, grey bar: Gelatin; **B) mechanical stability of alginate and alginate hybrid gels containing varying concentrations of PEG 300,000 and HA.** Addition of higher concentrations PEG, HA and the combination of 0.25% PEG and 0.2% HA resulted in significant lower mechanical stability compared to alginate gels without addition of HA or PEG (Alg2) (n = 3 ± SD; * p< 0,05; **p<0,01; ***p<0,001).

Since gelation induced only by either “internal” or “external” gelation alone did not provide gels that solidify in the desired time frame, we decided to combine the two methods (external + internal gelation). We added CaCO_3_ and GDL at a ratio of 2:1 to different concentrations to 2% alginate solutions (internal gelation) and further added CaCl_2_ solution (external gelation) to increase gelation speed. The mechanical strength of the formed alginate gels was measured directly after preparation and also after one week of storage. For all samples 200 mM CaCl_2_ solution was used, which had previously been determined as the optimal Ca^2+^ concentration for the external gelation (Fig. 2). As shown in Fig. 3, the addition of 50 mM CaCO_3_ results in a full gelation of the gels within 30 minutes, and the prepared gels show a compression force of 20 N on average. However, storage of the gels for one week resulted in a significantly decreased mechanical stability for all gels prepared with 50 mM CaCO_3_ or less. Essentially smaller changes were observed when the gels were prepared with 100 mM to 200 mM CaCO_3_. For these samples, the required compression force directly after preparation and after one week of storage remained almost constant (Fig. 4).

#### Effect of the chosen alginate on gel properties

We investigated two different alginates to study the effect of alginate composition on the matrix properties (Table 1). Alginate 1 was a synthetic alginate, whereas alginate 2 was an extract from brown algae. For alginate 2, two different batches were used to check for batch-to-batch variations. Gels were prepared as described before. In addition, PEG 300,000 and sodium hyaluronate (HA) were added to some samples, forming alginate hybrid gel. The mechanical properties were investigated as described before. Gels prepared from alginate 1 required lower compression forces compared to alginate 2. Between the two batches of alginate 2, no significant differences were observed. As a consequence, alginate 2 was used for further studies. For the alginate hybrid gels a similar trend was observed. For the formulations containing alginate 1 and the adhesive polymers (PEG, HA) lower compression forces were observed compared to the same gels prepared with alginate 2.

**Table 1:** Comparison of two different qualities of alginate and their impact on mechanical stability.

Formulation	Required force for 8 mm compression [N]	Standard deviation
**Alginate 1**	19.18	± 2.63
**Alginate 1 PEG 0.1% HA 0.1%**	18.93	± 1.37
**Alginate 2 batch 1**	33.28	± 5.07
**Alginate 2 batch 2**	33.80	± 5.89
**Alginate 2 PEG 0.1% HA 0.1%**	31.17	± 6.20

Alginate 1 (sodium alginate Biochemica) as a synthetic alginate, Alginate 2 (Alginic acid sodium salt from brown algae) as natural alginate. Three gels of each formulation were measured.

#### Enhanced adhesion to collagen

To improve adhesion properties of alginate gels to collagen, we systematically investigated the formulation of alginate hybrid gels by the addition of adhesive polymers. PEG with a molecular weight of 300,000 Da and HA having an intrinsic viscosity of 2.7 m^3^/kg were added to the 2% alginate gels prepared using the combination of internal and external gelation at different concentrations (Table 2). For all samples, 200 mM CaCl_2_ was used in addition for the external gelation, and 150 mM CaCO_3_ for the internal gelation. PEG 300,000 was chosen due to its reported bioadhesive properties [11]. The adhesive properties where then investigated using three different model surfaces: Teflon as an example for a surface with minimal adhesiveness, polystyrene as a polymer with some adhesiveness and gelatine gels as a model of biological origin. A schematic of the adhesion force testing is provided in Fig. 1b. The addition of PEG resulted in slightly increased adhesive forces to all test surfaces, as addition of sodium hyaluronate promoted adhesion to gelatine (Fig. 4a). Furthermore, we found an additive effect of both polymers on adhesion properties. Alginate hybrid gels containing 0.1% PEG and 0.1% HA or containing 0.25% PEG and 0.2% HA showed the best adhesive properties to the three different surfaces.

**Table 2:** Composition of alginate hybrid gel formulations.

Abbreviation	Alginate concentration [%]	PEG 300,000 concentration [%]	Hyaluronic acid (HA) concentration [%]
**Alg 2%**	2	--	--
**Alg 2% PEG 0.1**	2	0.1	--
**Alg 2% PEG 0.25**	2	0.25	--
**Alg 2% PEG 0.5%**	2	0.5	
**Alg 2% HA 0.1**	2	--	0.1
**Alg 2% HA 0.2**	2	--	0.2
**Alg 2% HA 0.4**	2	--	0.4
**Alg 2% PEG 0.1 HA 0.1**	2	0.1	0.1
**Alg 2% PEG 0.25 HA 0.2**	2	0.25	0.2

However, the addition of either PEG or HA decreased the mechanical stability of alginate gels in a dose dependent matter (Fig. 4b). Of the tested concentrations, only the addition of 0.1% PEG and 0.1% HA to the 2% alginate gel sufficiently improved adhesion to gelatine while keeping mechanical stability of the gel unaltered.

#### Sufficient mechanical stability for 6 weeks

**Fig 5:**
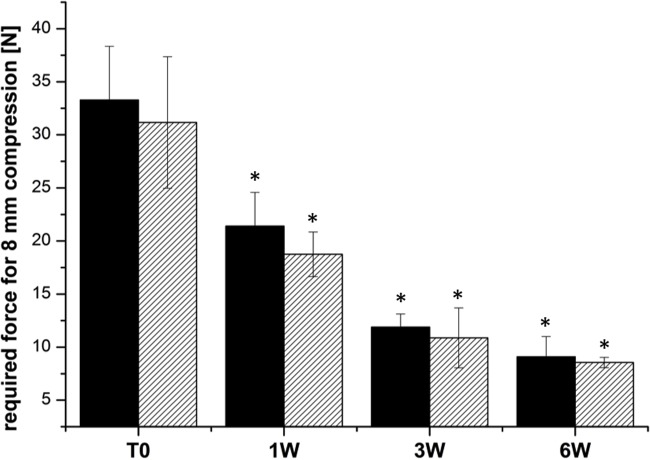
Mechanical stability of alginate gel (black bar) and alginate hybrid gel containing 0.1% PEG 300,000 and 0.1% HA (dashed bar) over a period of 6 weeks. All gels were stored in PBS. During aging, all gels show significant lower mechanical stability at 1, 3 and 6 weeks compared to day 0 (T0) (n = 3 ± SD; * p< 0,05; **p<0,01; ***p<0,001).

The mechanical stability of the alginate gels containing alginate or the alginate hybrid gel containing 0.1% PEG and 0.1% HA was investigated for a period of 6 weeks, the time frame required for healing after surgical intervention. Alginate gels incubated in PBS solution showed a decrease in mechanical stability upon storage. This decrease was observed for the alginate gel as well as for the alginate hybrid gel (Fig. 5). However, the shape of the gel did not change and the gel provided sufficient mechanical stability for the suspended cells.

#### Water uptake capacity of alginate hydrogels

The mass of the alginate hydrogels was weighted directly after preparation and 1, 7, 14, and 20 days after preparation to investigate whether the incubation in DMEM leads to a swelling of the hydrogels over time due to water uptake. No significant water uptake into alginate gels was observed after incubation in DMEM high glucose for 20 days (data not shown).

#### Injectability through a standard syringe

**Fig 6:**
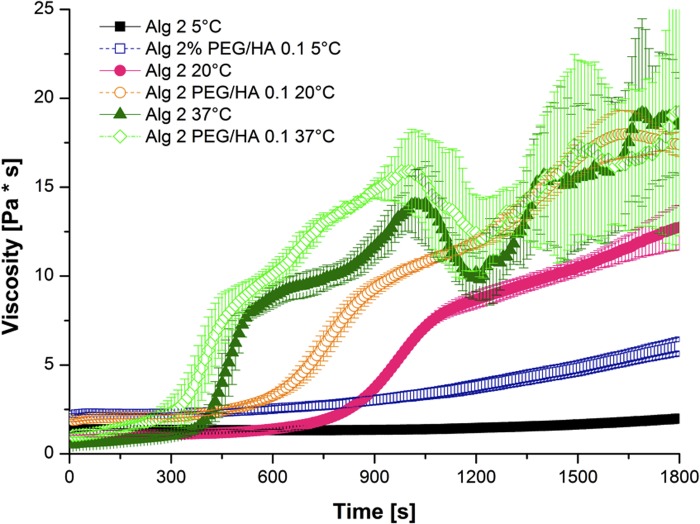
Viscosity of alginate and alginate hybrid gels investigated at 5°C, 20°C and 37°C as a function of time (n = 3 ± SD).

Of utmost important for the applicability of the investigated gels is not only their rapid gelling and mechanical stability, but also their injectability through a standard syringe, making the gels applicable for use in an arthroscopic setting. Rheological investigation of 2% alginate gels with 200 mM CaCO_3_ were carried out at three different temperatures: 5°C, 20°C and 37°C immediately after compounding. The results reveal that the alginate gels showed sufficient injectability for 10 minutes through a standard syringe at room temperature (Fig. 6). As expected, increasing the temperature resulted in faster gelation, whereas lower temperature lead to almost no gelation of the gels as the measured viscosity stayed low. Alginate hybrid gels with 0.1% PEG and 0.1% HA showed a faster gelation speed for all investigated temperatures. Optical observation of gel spreading after injection confirmed injectability of 2% alginate gels with addition of 0.1% PEG and 0.1% HA (data not shown).

#### Diffusion of cytokines from the stem cells through the gel matrix

**Fig 7:**
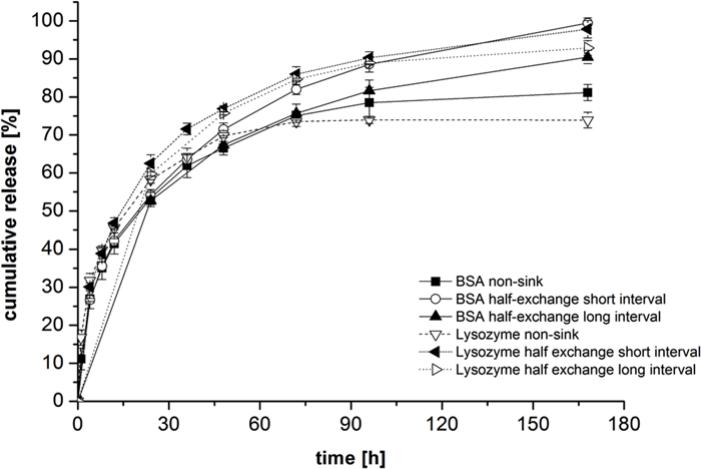
Cumulative release of BSA or lysozyme from alginate hybrid gels. Investigations were carried out using non-sink conditions, half-exchange within short intervals or half-exchange within long sampling intervals (n = 3 ± SD).

We performed diffusion experiments using the two model proteins BSA and lysozyme. The model proteins were added to alginate hybrid gels at a concentration of 5.0 mg/ml. Protein diffusion into the supernatant was measured after gelation of the gels by UV spectrophotometry. Three different conditions were used to investigate potential sampling effects on the release: 1) non-sink conditions (black square), half buffer exchange with short intervals (open circle), half buffer exchange with long intervals (black diamond). The experiment revealed good permeability through the alginate gels for both model proteins, irrespective of the employed sampling mode (Fig. 7).

### Evaluation of alginate gels loaded with primary human mesenchymal stem cells

#### Gel composition and cell viability

**Fig 8:**
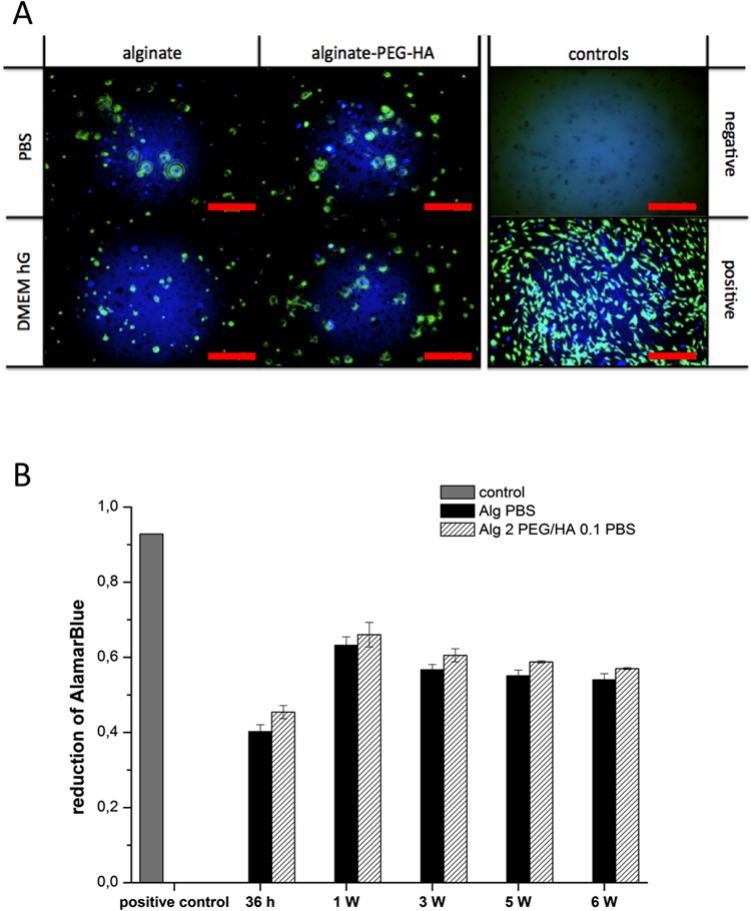
Viability of MSCs within alginate gels. **A) Fluoresence microscopy of alginate gel, alginate hybrid gel and controls after 6 weeks of incubation.** Representative pictures of three gels of each formulation, each loaded with MSCs from different donors. CalceinAM is displayed in green. Hoechst staining is displayed in blue. Samples were investigated in PBS and DMEM containing high glucose (DMEM hG). Negative control: alginate gel without cells; positive control: MSCs in 2D culture on plastic dishes **B) Percent reduction of Alamar Blue in the in vitro cell culture using human MSCs.** Positive control: MSCs in 2D culture (grey bar), alginate gel (black bar) and alginate hybrid gel containing 0.1% PEG and 0.1% HA (dashed bar) are displayed (n = 3 ± SD).

The next important aspect was to analyse the impact of gel composition on cell viability. First of all, the suspension of primary human mesenchymal stem cells within different 2% alginate gels resulted in homogenous distribution of the cells within the matrix after gelation. Internal vs. external gelation and the addition of 0.1% PEG and 0.1% HA had no significant negative impact on cell viability (Fig. 8a). During the 6 weeks culture period, cell number slowly increased, as visible in light microscopic investigation (data not shown). Viability testing of the cells with Alamar blue assay confirmed that the cells remained viable within the gels for at least 6 weeks. However, due to the limited diffusion capacity of Resazurin from the gels, the Alamar blue assay was only sufficient to provide qualitative and not quantitative data on cell viability (Fig. 8b). After 6 weeks of culture, the gels were homogenously colonized with viable cells. Alamar blue testing of the culture dishes at each time point revealed that cells were sufficiently immobilized within the gels and only a marginal amount of cells migrated out of the matrix (data not shown).

#### Gel composition and growth factor release

**Fig 9:**
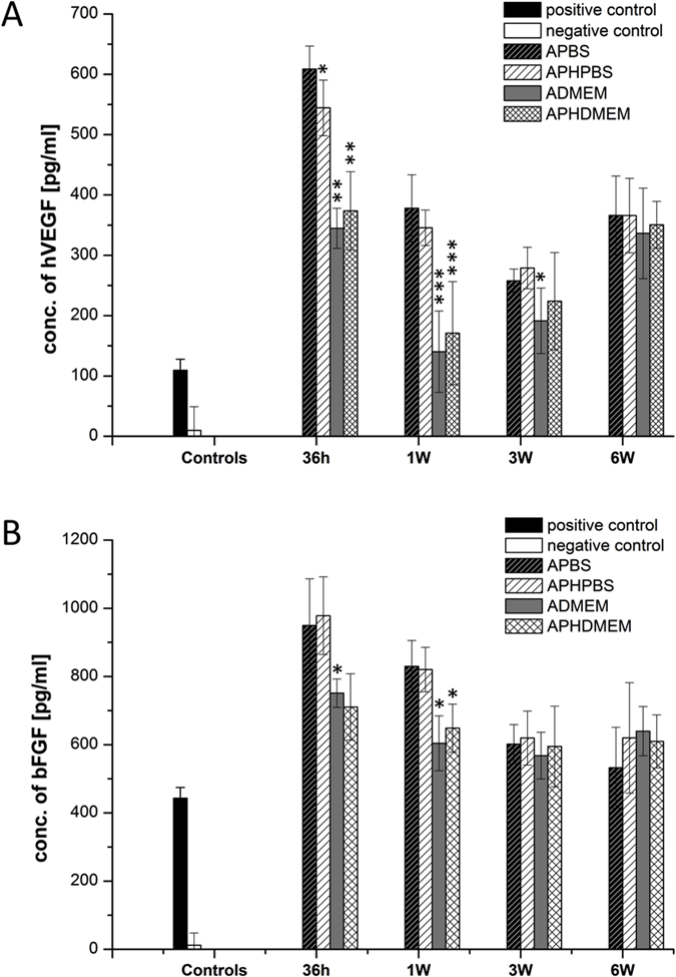
Concentration of released growth factors. **A) hVEGF and B) bFGF in MSC cell culture supernatants determined over a period of 6 weeks.** Positive control (black bar), negative control (white bar). Alginate gel in PBS (APBS) dark dashed bar, alginate hybrid gel (APHPBS) light dashed bar, alginate gel in DMEM (ADEMEM) grey bar, alginate hybrid gel in DMEM (APHDMEM) crossed bar. MSCs seeded into gels containing DMEM partially show significant lower growth factor release during the 6 week investigation, if compared to MSCs in alginate gels containing PBS (APBS) at the same time point (n = 3 ± SD; * p< 0,05; **p<0,01; ***p<0,001).

As proof of concept, human mesenchymal stem cells were cultured within alginate gels (2% Alginate + 0.1% PEG + 0.1% HA) for 6 weeks and the release of anabolic growth factors VEGF (Fig. 9a) and bFGF (Fig. 9b) from the matrix was monitored. In order to simulate physiological conditions as close as possible, 2% alginate gels and 2% alginate hybrid gels containing 0.1% PEG 300,000 and 0.1% HA were prepared in PBS and in comparison also in cell culture media (DMEM). As confirmed by ELISA a high paracrine activity of the immobilized mesenchymal stem cells was observed for 6 weeks. Robust release of VEGF and bFGF from the matrix/cell-constructs could be detected during the whole observation period. Here, the addition of 0.1% PEG and 0.1% HA had no significant influence on growth factor release. Interestingly, hVEGF and bFGF release from alginate gels and alginate hybrid gels prepared with DMEM were lower compared to the samples prepared with PBS.

## Discussion

In an effort to make the paracrine activity of autologous MSCs usable to enhance tissue regeneration after surgery, we designed a matrix that allows immobilization of MSCs harvested during operation in the operation situs. One reported option to accelerate healing processes is the administration of stem cells directly into the surgical wound or onto the injured fibre. It has been reported that particularly stem cells have the ability to differentiate into a variety of tissue cell types [12,13]. MSCs expanded *ex vivo* differentiated into cells of the residing tissue, were able to repair the damaged tissue, and partially restored it to its normal function [12]. In addition, the release of a variety of signals such as growth factors (IGF-1, HGF, VEGF, IGF-2 or bFGF) and cytokines assist in the healing process [14]. One challenge, however, is to provide an extracellular matrix system which displays sufficient mechanical stability and provides an environment in which the cells survive. As basis for this matrix we used alginate. Alginate hydrogels show a good biocompatibility and are relatively inert, when administered to animals [15]. Here, studies revealed that a high grade of purity is necessary to avoid inflammatory responses of the recipient, which may be induced by contaminants [16].

We found that the addition of only low concentrations of calcium ions (200 mM CaCl_2_) is sufficient to initiate the cross-linking process and the induction of gel formation (“external gelation”). These concentrations of divalent ions have been reported to be non-detrimental to cells [17]. To allow faster gelation and enhanced long-term stability, we added CaCO_3_ to the alginate gels as internal source of calcium (“internal gelation”). CaCO_3_ has been proven to be inert and biodegraded, when implanted [18]. By combining internal and external gelation, we were able to delay the degradation of the alginate matrix *in vitro*. Fast degradation of alginates is one of the major problems in cell therapy and tissue engineering applications [16,19].

We have added hyaluronic acid and PEG to the alginate gels to improve adhesion to gelatin, which served as a model for a biological surface. Both PEG and HA are known to be biocompatible after implantation [20]. Addition of PEG and HA did additively improve adhesion forces. The low concentrations used in our gels did not significantly decrease mechanical stability or increase toxicity to cells. This is in line to previous published results using PEG to enhance adhesive properties of alginate gels [21]. The achieved adhesion properties are a prerequisite to allow application of the gels by a syringe in a minimal invasive procedure, as no further fixation of the gels is necessary.

The hydrogel scaffolds were designed to enhance healing after surgery of soft tissues. Here, a scaffold of low mechanical stiffness is favorable to avoid mechanical irritation on the surgical site. On the other hand, a sufficient mechanical stability is necessary to realize an application in musculoskeletal tissues, where mechanical forces act on the hydrogel. Here, alginate hydrogels show advantages, as they can be simply adapted to the desired stiffness, changing the alginate concentration [22]. Our study shows that alginate gels containing adhesive polymers prepared by a combination of internal and external gelation are injectable through a standard syringe, show sufficient mechanical properties, form a microenvironment in which stem cells survive, and allow for cytokine diffusion.

The used alginate/hyaluronic acid/PEG hybrid gels successfully immobilize human mesenchymal stem cells for at least 6 weeks. This is in line to previously published results for alginate beads used to immobilize cells for cell therapeutic applications [23]. Cells encapsulated within the hydrogels showed robust viability during that time period in our experiment. This is remarkable, as failure of cell therapeutic approaches using alginate gels was interpreted as a result of the hypoxia within the hydrogels [24]. However, a mild hypoxia within the hydrogels may even be desired in our approach. It could stimulate the immobilized MSCs to produce and secrete higher amounts of VEGF into the surrounding tissue [25].

We could prove that the used alginate/hyaluronic acid/PEG hybrid gels are permeable for growth factors released by the incorporated mesenchymal stem cells. This is in line with several studies investigating alginate hydrogels as drug delivery system. Robust secretion of growth factors VEGF and bFGF out of the MSC loaded hydrogels was detectable in the supernatant. Here, bFGF concentrations were larger 500pg/ml for at least 6 weeks. This is equivalent to at least 2 times the ED50 reported for bFGF [26]. In contrast, VEGF concentrations only reached half ED50 reported for that cytokine [27]. However, the present *in vitro* experiments were carried out at ambiguous atmosphere. Here, oxygen partial pressure is much higher than in the capillaries and tissues of the body [28]. As MSCs secrete VEGF dependent to oxygen partial pressure, it is likely, that immobilized MSCs will secrete higher amounts of VEGF when hydrogels are implanted at physiological conditions [25].

In our experimental setting, a number of 3x10^6^ stem cells were used to load the gels. This amount was used, as it represents the amount of cells usually obtained from a 175cm^2^ cell culture flask. However, the ideal amount of stem cells to immobilize within the alginate gels remains unclear. To determine this, further studies—including *in vivo* investigations—will be necessary.

Within the present work, we have established a method that makes the paracrine activity of MSCs usable to enhance healing after surgery. Several studies confirmed the possibility to enhance regeneration after surgery by local use of anabolic growth factors [1,3,4,5,29]. Here, a long-term application was favorable [30]. This could either be achieved by repetitive injection of recombinant growth factors or viral transfection. Both methods are not transferable to the patient. The presented method could allow local application of anabolic growth factors for at least 6 weeks. It could be directly transferred to the operation room, as it is designed for single step application in open and arthroscopic surgeries. Furthermore, the safety of application of autologous mesenchymal stem cells could be demonstrated in a number of studies as reviewed by [7]. Therefore, the presented method could be transferred to clinic as a cost effective and safe single step method to enhance healing after a broad variety of surgeries.

## Conclusion

The presented *in vitro* results are an encouraging proof of principle that alginate gels are a suitable matrix for stem cells. Now the effectiveness of this system has to be proven *in vivo*. The gels can be a valuable tool to make the paracrine MSC activity usable for enhanced tissue regeneration after surgery.
